# Faecal metabolite deficit, gut inflammation and diet in Parkinson's disease: Integrative analysis indicates inflammatory response syndrome

**DOI:** 10.1002/ctm2.1152

**Published:** 2023-01-01

**Authors:** Aisha Augustin, Adrien Le Guennec, Chianna Umamahesan, Aidan Kendler‐Rhodes, Rosalind M. Tucker, Elena Chekmeneva, Panteleimon Takis, Matthew Lewis, Karthik Balasubramanian, Neville DeSouza, Benjamin H Mullish, David Taylor, Suzanne Ryan, Kevin Whelan, Yun Ma, Mohammad A. A. Ibrahim, Ingvar Bjarnason, Bu’ Hussain Hayee, André Charlett, Sylvia M. Dobbs, R. John Dobbs, Clive Weller

**Affiliations:** ^1^ Institute of Pharmaceutical Science King's College London London UK; ^2^ The Maudsley Hospital London UK; ^3^ NMR Facility King's College London London UK; ^4^ Nutritional Sciences King's College London London UK; ^5^ National Phenome Centre Imperial College London London UK; ^6^ Section of Bioanalytical Chemistry Imperial College London London UK; ^7^ Department of Metabolism Digestion and Reproduction Imperial College, London UK; ^8^ Imaging King's College Hospital London UK; ^9^ Institute of Liver Studies King's College Hospital London UK; ^10^ Immunological Medicine King's College Hospital London UK; ^11^ Gastroenterology King's College Hospital London UK; ^12^ Statistics, Modelling and Economics UK Health Security Agency London UK

**Keywords:** faecal metabolome, intestinal inflammation, Parkinson's disease, systemic immunome

## Abstract

**Background:**

Gut‐brain axis is widely implicated in the pathophysiology of Parkinson's disease (PD). We take an integrated approach to considering the gut as a target for disease‐modifying intervention, using continuous measurements of disease facets irrespective of diagnostic divide.

**Methods:**

We characterised 77 participants with diagnosed‐PD, 113 without, by dietary/exogenous substance intake, faecal metabolome, intestinal inflammation, serum cytokines/chemokines, clinical phenotype including colonic transit time. Complete‐linkage hierarchical cluster analysis of metabolites discriminant for PD‐status was performed.

**Results:**

Longer colonic transit was linked to deficits in faecal short‐chain‐fatty acids outside PD, to a ‘tryptophan‐containing metabolite cluster’ overall. Phenotypic cluster analysis aggregated colonic transit with brady/hypokinesia, tremor, sleep disorder and dysosmia, each individually associated with tryptophan‐cluster deficit. Overall, a faster pulse was associated with deficits in a metabolite cluster including benzoic acid and an imidazole‐ring compound (anti‐fungals) and vitamin B3 (anti‐inflammatory) and with higher serum CCL20 (chemotactic for lymphocytes/dendritic cells towards mucosal epithelium). The faster pulse in PD was irrespective of postural hypotension. The benzoic acid‐cluster deficit was linked to (well‐recognised) lower caffeine and alcohol intakes, tryptophan‐cluster deficit to higher maltose intake. Free‐sugar intake was increased in PD, maltose intake being 63% higher (*p* = .001). Faecal calprotectin was 44% (95% CI 5%, 98%) greater in PD [*p* = .001, adjusted for proton‐pump inhibitors (*p* = .001)], with 16% of PD‐probands exceeding a cut‐point for clinically significant inflammation compatible with inflammatory bowel disease. Higher maltose intake was associated with exceeding this calprotectin cut‐point.

**Conclusions:**

Emerging picture is of (i) clinical phenotype being described by deficits in microbial metabolites essential to gut health; (ii) intestinal inflammation; (iii) a systemic inflammatory response syndrome.

## Graphical abstract

A marker of intestinal inflammation was 44% greater in Parkinson's disease, exceeding a cut‐point for inflammatory bowel disease in 16%. It was associated with a higher maltose and sucrose intake. The emerging picture is of systemic inflammatory response consequent on intestinal inflammation, clinical phenotype being described by deficits in faecal metabolites essential to gut health.

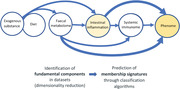

## 1 BACKGROUND

Parkinson's disease (PD) is traditionally characterised by brady/hypokinesia, rigidity, tremor and postural instability. However, absence of overall delay in colonic transit and of transverse colon segmental delay, irrespective of laxative use, had 90% specificity for excluding PD.^1^

We have reported decreased frequency of defecation for up to three decades before neurological diagnosis, with worsening after diagnosis.^2^ Metanalysis^3^ and primary care data mining^4^ show that constipation doubles the risk of PD‐diagnosis 10 years on. Outside diagnosed‐PD, lower defecation frequency has been associated with decreased substantia nigral neuronal density and increased Lewy bodies (pathological hallmark of PD) in post‐mortem brain. Post‐diagnosis, worsening constipation correlated with nigrostriatal dopaminergic neuronal loss on functional imaging.^5^

Colonic Lewy body pathology is present 2 to 5 years before PD‐diagnosis.^6^ It has been associated with human colitis and with chemically induced rodent colitis.^7^ In PD patients with constipation, chronic colitis with CD4+ cell mucosal infiltration is reported.^8^ Messenger RNA expression of pro‐inflammatory cytokines and glial markers has been found elevated in ascending colon biopsies from PD patients compared with controls.^9^ The risk ratio for PD in either Crohn's disease (CD) or ulcerative colitis (UC) is reported as 1.4.^10^ The hazard ratio given for incident PD is 3.5 times greater in microscopic colitis (first 2 years post‐biopsy) than in the general population, that is, similar to the odds of having microscopic colitis in established PD.^11^ Two genetic risk factors for inflammation (mutations in LRRK2 and in NOD2) occur in both CD and PD.^10^ However, the PD‐related mutation G2019S increases LRRK2 kinase activity, while the IBD risk allele M2397 affects LRRK2 accumulation but not kinase activity.^12^ The role of NOD2 in controlling inflammation, by maintaining equilibrium between intestinal microbiota, mucosa and host immune responses, is lost with mutations.^13^ Single‐gene inborn errors of immunity, associated with chronic intestinal inflammation, are implicated as PD‐associated polymorphisms [interleukin (IL)‐10, IL‐10RA, IL‐10RB].^14^

Parkinson's disease could fit with the concept of premature or accelerated ‘inflammaging’ (chronic, low‐grade inflammation developing with advanced age, in the absence of overt infection).^15^ For example, the serum concentration of the pro‐inflammatory cytokine, IL‐6, is predictive of incident PD^16^ and its increment with age in PD is equivalent to more than a decade of premature ageing.^17^ Moreover, there are biological gradients of objective measures of PD‐facets on blood leucocyte subtype counts and circulating inflammatory markers.^17^, ^18^, ^19^, ^20^ PD manifestations could represent a *forme fruste* of systemic inflammatory response syndrome (SIRS).^21^ Neuroinflammation, consequent on systemic, would be in response to intestinal inflammation and barrier translocation of microorganisms/their products.^10^ In early PD, neuroinflammation (imaging microglial activation) accompanies neuronal damage (loss of presynaptic dopamine transporter).^22^ Histopathologically, it persists as the proportion of IgG‐labelled nigral neurons decreases,^23^ suggesting that neuroinflammation is driving neuronal death, rather than just being reactive to aberrant protein deposition or degenerating neurons.

This is a hypothesis building study of the pathophysiology of PD, central to which is a non‐targeted exploration of faecal metabolome using ^1^H‐nuclear magnetic resonance (NMR) and measurement of intestinal inflammation by faecal calprotectin. We integrate nutrient and medication intake, systemic immunome and clinical phenotype including colonic transit.

## 2 MATERIALS AND METHODS

### 2.1 Participant groups

This observational study was set in a National gut‐brain axis research clinic with a focus on quantifying disease facets to define temporal and interventional change. People with diagnosed‐PD were invited to volunteer. Inclusion was according to UK Brain Bank Criteria with at least three supportive criteria,^24^ after exclusion of causes of secondary parkinsonism. Evidence of responsiveness to levodopa was not required. Probands’ cohabiting life partners were also invited to enlist. ‘Controls‐proper’ neither had diagnosed‐PD nor resided with anyone who did. Website recruitment was complemented by proband dissemination, the latter facilitating control recruitment. The study was approved by King's College London Research Ethics Committee, with participants giving written informed consent. All participants underwent subjective and objective assessments of clinical phenotype on recruitment. Table 1 contrasts demographic, nutritional and phenotypic characteristics of, and an intestinal inflammation marker in, 77 participants with diagnosed‐PD and the ‘remainder’ 113 without including 53 probands’ life partners.

**TABLE 1:** Participant characteristics

Characteristics	Median (lower, upper quartile)^†^ at first assessment	
	PD	Remainder
	(*n* = 77)	(*n* = 113)
**Demographic**		
Age (years)	70 (62, 74)	67 (59, 70)
Sex (male)	60^†^	42^†^
Height (cm)	170 (161, 178)	169 (162, 178)
Weight (kg)	72 (63, 84)	71 (63, 82)
Body mass index (kg/m^2^)	24.7 (22.4, 28.9)	24.4 (22.6, 26.7)
Time since diagnosis (years)	8.5 (5, 14)	
Laxatives (yes)	59^†^	12^†^
Anti‐parkinsonian medication (yes)	77^†^	0^†^
Proton‐pump inhibitor (yes)	21^†^	9^†^
Non‐steroidal anti‐inflammatory drug (yes)‡	16^†^	9^†^
Number of antimicrobial courses in last 3 years	1 (0, 2)	1 (0, 1)
Time since last antimicrobial course (years)	0.5 (0.2, 1.5)	0.9 (0.3, 1.5)
Total life‐time experience of tobacco smoking (years)	2 (0, 15)	0 (0, 12)
Time since tobacco smoking abandoned (years)	38 (29, 48)	36 (22, 44)
**Nutrient intake including supplements (5‐day diary)**		
Energy intake (kcal/day)	1768 (1552, 2133)	1801 (1518, 2077)
Carbohydrate (g/day)	199 (170, 243)	186 (144, 223)
Free sugars (g/day)	32 (23, 44)	25 (19, 37)
Fat (g/day)	71 (61, 100)	73 (55, 85)
Cholesterol (mg/day)	229 (165, 307)	219 (166, 271)
Protein (g/day)	68 (59, 85)	72 (59, 87)
Fibre (g/day)	21 (18, 24)	20 (17, 25)
Alcohol (g/day)	3.1 (0.1, 10.5)	12.6 (2.5, 19.3)
Caffeine (mg/day)	116 (78, 196)	174 (128, 243)
Water (ml/day)	2082 (1758, 2503)	2329 (1932, 2814)
** Hypokinesia**		
Mean stride length at free walking speed (cm)	113.4 (96.7, 124.1)	127.5 (116.7, 135.2)
** Rigidity** (torque for passive extension of forearm at elbow)		
Flexor rigidity on worse side (Nmx10^–3^ ^10–3^)	496 (332, 766)	259 (128, 388)
** Tremor**		
Rest tremor (detectible)	77^†^	35^†^
** Postural abnormality**		
UPDRS^§^ motor subscore 3.13 (normal, 0; severe 4)	1 (0, 2)	0 (0, 0)
**Psychomotor and psychometric**		
Unwarned reaction time (ms)	685 (577, 825)	607 (555, 680)
Warned reaction time (ms)	481 (358, 619)	412 (345, 474)
Cognitive processing time (ms)	212 (133, 291)	199 (149, 243)
Mini‐mental score	30 (28, 30)	30 (30, 30)
Depression score	11 (6, 15)	4 (1, 9)
Anxiety score	11 (6, 18)	4 (2, 7)
Rapid‐eye‐movement sleep disorder score	4 (3, 7)	2 (1, 4)
**Blood pressure and pulse**		
Mean arterial pressure (MAP) lying (mmHg)	92 (84, 102)	94 (87, 102)
MAP immediate standing (mmHg)	86 (78, 95)	93 (86, 100)
MAP 1 min standing (mmHg)	89 (80, 96)	95 (88, 102)
MAP 3 min standing (mmHg)	89 (80, 96)	96 (87, 102)
Pulse lying (/min)	68 (60, 75.5)	63 (57, 68)
Pulse immediate standing (/min)	77 (67, 84)	71 (60, 79)
Pulse 1 min standing (/min)	76 (68, 83)	72 (64, 80)
Pulse 3 min standing (/min)	77 (66, 82)	72(65, 80)
**Bowel function**		
Total number of markers retained	20 (11, 36)	10 (6.5, 16.5)
Number of markers retained in transverse colon	4 (1, 8)	2 (1, 4)
Rome functional constipation (yes)	31^†^	25^†^
Bristol Stool Scale	3 (2, 4)	3 (2, 4)
** Intestinal biomarkers**	**Median concentration (5, 95 percentile)** (number observations including follow‐up)	
	**PD**	**Remainder**
Faecal calprotectin (μg/g)	34 (6, 393)	19 (7, 136)

^†^Exceptions as percentages.

^‡^Aspirin, excluded when prescribed in low‐dose as anti‐platelet treatment.

^§^Unified Parkinson's Disease Rating Scale.

### 2.2 Clinical phenotyping and nutrient intake assessment

#### 2.2.1 Clinical assessment

Cardinal motor features of PD were assessed as follows: Brady‐ and hypokinesia was measured by free walking speed and mean stride length.^25^ Rigidity was measured as torque required of an electric motor to move the relaxed supported forearm horizontally through a 40˚ arc about the elbow, at a controlled velocity.^26^ Tremor was recorded as observed, or not, under the following conditions: at stance and during walking, interview and mental agility testing (repeating incremental series of digits, in reverse order). Posture was scored according to the Unified Parkinson's Disease Rating Scale (UPDRS)^27^ motor subscore 3.13 [normal (0) to severe (4)], based on the worst posture observed standing and walking, and ability to correct posture.

Psychometric assessment was by (i) questionnaires: mini‐mental state examination (out of 30), depression and anxiety inventories (each out of 63) and rapid‐eye‐movement sleep behaviour disorder (out of 12, i.e. not scoring for the item presence/absence of ‘nervous system’ disease)^28^, ^29^, ^30^, ^31^ and (ii) efficiency of cognitive processing, as measured objectively by shortening of reaction time in response to a prompt in the alerting signal.^32^

Orthostatic cardiovascular responses were summarised as mean arterial pressure, lying and standing (immediate and after 1 and 3 min), and corresponding pulse rates.

Bowel function was defined by (i) questionnaires: Rome III functional constipation for its presence/absence,^33^ and Bristol Stool Scale for consistency of stool (1–7),^34^ (ii) colonic transit time using pellets containing radio‐opaque barium markers (Transit‐Pellets, Medifactia, Stockholm, Sweden), swallowed on 6 consecutive days.^1^ An abdominal radiograph was taken on day 7, approximately 12 h after the last pellet. All radiographs were assessed (blind to participant identity/group) by the same specialist gastroenterological radiologist.

Current consumption of proton‐pump inhibitors (PPIs) and non‐steroidal anti‐inflammatory drugs was recorded, as was number of antimicrobial courses, in the last 3 years, and time since the last course.

#### 2.2.2 Nutritional intake

Intake was measured using a standard consecutive 5‐day unweighted food, drink and supplement diary. Participants were provided with detailed verbal and written instructions on how to complete the diaries. It was explained how to estimate portion size using household measures (e.g. teaspoons, cup sizes). Validated coloured photographs were provided as examples of how to estimate portions of composite dishes. Participants were asked to maintain their usual intake for the duration of the diary. Participants with PD who had difficulty completing the food diaries were aided by their partner or carer. Participants were telephoned, initially to remind them of the importance of adequate completion, and, on completion of the diary, for clarification where necessary.

Dietary data were entered onto an online nutritional analysis software (Nutritics, Research Edition, v5.021; Dublin). The database contains over 125 000 foods, with reference to the UK Composition of Foods database. Data were entered by participant number and visit‐day code, separately by two analysts.

### 2.3 Sample collection and processing

Faecal samples were collected by participants prior to arrival, or on‐site, using a standardised kit, previously posted with instructions stressing that delivery must be within 4 h of evacuation (time noted). Participants unable to comply with instructions that day did so in the subsequent few days. Faecal aqueous extracts^35^ were obtained from one sample tube per person, stored in aliquots of ≥0.5 ml. Blood was collected in clot activator/serum separating tubes, and allowed to stand for 15–30 min, before centrifuging at 4°C for 15 min at 2500 × *g*. Serum was stored in 250 μl aliquots. All samples were frozen at –80°C directly after processing.

### 2.4 Faecal metabolome

Each thawed faecal aqueous extract (500 μl) was mixed with 100 μl of heavy water, D_2_O, containing 0.05% w/w 3‐(trimethylsilyl)propionic‐2,2,3,3‐D_4_ acid (TSP) and 2% sodium azide, and buffered to pH 7.4 with 100 mM phosphate. ^1^H NMR spectra were obtained on a Bruker AVANCE 700 spectrometer (Billerica, Massachusetts), at proton frequency 700 MHz, using a Carr‐Purcell‐Meiboom‐Gill pulse sequence. Acquisition in automation used TopSpin v.4.0 (Bruker, BioSpin AG). Pre‐processing entailed Fourier transformation, phase adjustment, baseline correction and calibration by reference to peak TSP resonance at δ = 0 ppm.

Spectral data were imported to MATLAB R2018b (Mathworks, Natick, Massachusetts) using a custom workflow toolbox (Edison NMR metabolomics laboratory, University of Georgia, USA). Residual water peaks were removed (δ = 4.63–5.20 ppm), as were any polyethylene glycol (PEG) signals (δ = 3.53–3.85), attributable to macrogol laxatives. Samples (14) with gross spectral distortion due to PEG or (2) unusual NMR peaks were eliminated from exploration in the first 135 samples (44/119 were from PD‐probands). Peak alignment by fast Fourier transform^36^ was followed by probabilistic quotient normalisation,^37^ then scaling by log transformation.

Databases, using 1D and 2D NMR (see below), for assignment of endogenous faecal metabolites were Complex Mixture Analysis by NMR (Ohio State University, USA);^38^ Human Metabolome (https://hmdb.ca/); Human Faecal Metabolome (https://faecalmetabolome.ca/); Biological Magnetic Resonance Bank (https://bmrb.io); and Spectral Database for Organic Compounds (https://sdbs.db.aist.go.jp). These were supplemented by published literature (e.g. for homarine, https://doi.org/10.1093/ajcn/nqz293). Statistical spectroscopic methods, Statistical Total Correlation Spectroscopy (https://pubs.acs.org/doi/10.1021/pr050399w) and Small Molecule Enhancement Spectroscopy (https://doi.org/10.1039/D0SC01421D) were applied to full spectra to suppress any macromolecular background and facilitate metabolite identification.

2D NMR analysis was aimed at identifying ‘unknown’ metabolites, critical to ‘3.3. Metabolome signatures of disease facets' (see below). Aliquots (500 μl) of 15 aqueous extracts, containing higher amounts of signals of interest, were dehydrated (Eppendorf vacufuge concentrator) for 18 h, reconstituted in 120 μl D_2_O containing 0.05% w/w of TSP and 2% of sodium azide, and pooled to give three (600 μl) five‐sample clusters. Spectra were acquired over 72 h. A range of 2D NMR spectroscopy experiments were performed: J‐Resolved Spectroscopy; Total Correlation Spectroscopy (^1^H,^1^H‐TOCSY); and Heteronuclear Single Quantum Coherence (^1^H,^13^C‐HSQC). Where attribution was putative, 1D spectra were recorded before and after spiking samples with chemical standards [e.g. 500 μM homarine (Toronto Research Chemicals, North York, Ontario); 75 μM hypoxanthine (Sigma, St. Louis, Missouri)] to confirm assignment (see also Supporting Information).

### 2.5 Serum immune analytes

Multiplex‐assays (Human Magnetic Luminex Performance Assays, R&D systems, Minneapolis, Minnesota) were used for 22/23 serum immune analytes (see Supporting Information), associated in literature with PD/co‐morbidities.^39^ A sandwich enzyme‐linked immunosorbent assay (ELISA) (Quantikine High Sensitivity ELISA, R&D systems) was used for IL‐17. Exploration in the first 72 participants’ sera (assayed in duplicate) found acceptable intraclass correlations (≥0.9) and left censoring (<10%) for 8/23 analytes: chemokine (C‐C motif) ligand CCL20, epidermal growth factor, IL‐6, IL‐17, leptin, macrophage migration inhibitory factor, resistin and tumour necrosis factor (TNF)‐α.

### 2.6 Intestinal inflammation marker

Faecal calprotectin concentration was measured as a marker of colonic inflammation (Bühlmann Laboratories, Schönenbuch, Switzerland). Cut‐points of >50 μg/g^40^ for presence of histological activity and 188 μg/g^41^ for ‘clinically significant inflammation’ comparable with that found in IBD were selected, recognising both the diversity of recommendations and the importance of continuous numerical data in addressing inflammaging. Optimal standard curve fit for the enzyme‐linked immunosorbent assays (ELISA) was log concentration against log optical density. Intraclass correlation (95% CI) was excellent: 0.98 (0.97, 1.00). We included a second observation, 1 year after the first, to indicate trajectory.

### 2.7 Statistical analysis

Two steps towards constructing a pathophysiological model for PD were made: (i) Appropriate dimension reduction by identifying redundant components within each functional data set and (ii) Assessing strength of association of remaining components with participant group‐status and with phenotypic facets measured across the diagnostic threshold. Appropriate generalised linear regression models were used, age, sex, weight and body mass index being considered as potential confounders. Disease‐status interaction terms were included in regression models: while significant interactions are noted, we recognise that non‐significance may be explained by insufficient power. Variables exhibiting a positively skewed distribution were transformed to approximate normality using natural logarithms. Models were fitted within Stata 15.1 (StataCorp, College Station, Texas).

Regarding metabolite profile: (i) Ratio of mean ‘concentration’ in those with PD to that in remainder provided a summary comparison between groups across all 98 metabolites documented by one analyst: 36 of these were associated with PD [age, sex, couple status^42^ and false‐discovery rate adjusted (*q*‐value cut‐point <.05)]. ‘Concentration’ is taken as directly proportional to area under corresponding NMR peak. There was good agreement with output of an independent analyst. (ii) Complete‐linkage hierarchical cluster analysis (CL‐HCA), based on the L2 dissimilarity measure (i.e. Euclidian distance for metabolites), was visualised in dendrogram format. Optimal number of clusters was determined by greatest Calinski and Harabasz pseudo‐*F* index (i.e. ratio of between‐cluster to within‐cluster variance).^43^ Data redundancy was defined by selecting only those metabolites complementary, within cluster, in explaining PD‐status or major phenotypic correlates. Metabolite associations were explored before and after adjusting for stool consistency (participant's contemporaneous Bristol Stool Scale rating). Heatmaps were used to visualise strength of associations.

Regarding mixed categorical and continuous phenotype measures, CL‐HCA was based on Gower dissimilarity (https://doi.org/10.1002/9781118445112.stat05670.pub2), a solution for number of clusters being obtained using the Duda and Hart pseudo‐*T* ^2^ statistic.^44^

## 3 RESULTS

We begin by clustering faecal metabolites discriminant for PD‐status. We proceed to dimensionality reduction, before heatmapping to produce metabolic signatures of disease facets and examining the relationship between metabolome and dietary characteristics of PD. We, then, explore the circulating immunome in relation to disease facets and metabolome. We complete the unfolding picture by putting the findings in the context of intestinal inflammation, as described by faecal calprotectin.

### 3.1 Four faecal metabolite clusters discriminate for PD

**FIGURE 1:**
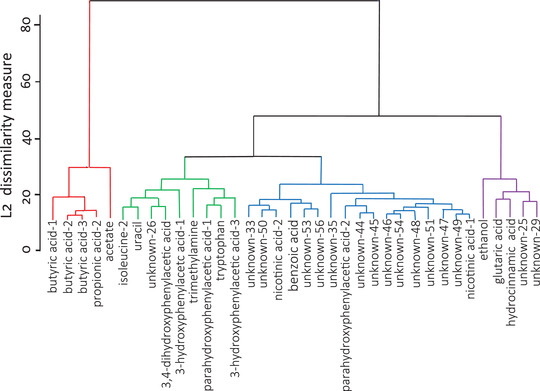
Dendrogram of faecal metabolites discriminant for PD. Four clusters of metabolite peaks were identified using the L2 dissimilarity measure: Cluster‐1 in red, Cluster‐2 green, Cluster‐3 blue, Cluster‐4 purple

Figure 1 demonstrates clustering of the 36 faecal metabolites individually discriminant for PD‐status (*q* value ≤ .05). The optimum number of clusters was four, with a pseudo‐*F* index of 134.89.

### 3.2 Dimensionality reduction

**FIGURE 2:**
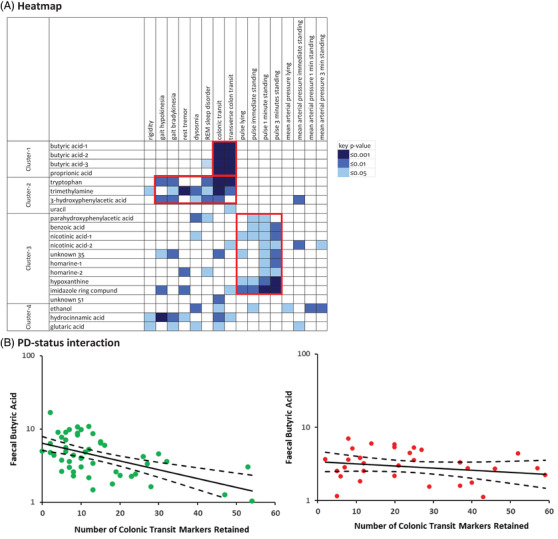
Faecal metabolite signatures. (A) Heatmap of metabolite concentrations against phenotypic measures irrespective of PD‐diagnostic threshold. Red boxes highlight areas of particular interest (Area‐1 at top, Area‐2 middle and Area‐3 bottom). Significance and directionality of effects (linear regression) remained after correction for stool consistency. Note: (i) Hydrocinnamic acid, Cluster‐4, has some similarity of facet associations to Area‐2 metabolites. (ii) Intestinally generated ethanol had a hypotensive effect. (B) Effects of faecal butyric acid concentration on total colon transit time according to PD‐status. Regression lines (95% CI) are shown, data points for participants without PD being in green, with PD in red. Number of markers retained in colon is used as an estimate of transit time. Chemical shift in butyric acid most markedly diminished in PD was analysed

Only faecal metabolite peaks complementary within cluster in discriminating for PD‐status were candidates for heatmapping against phenotype. Three chemical shifts for butyrate and two for nicotinic acid dominated their spectral regions and were complementary within cluster. That different shifts for each of these metabolites had similar behaviour in heatmapping gives confidence in attribution (Figure 2A). (The shift most diminished in PD was used in further analysis.) In contrast, although two peaks were attributed initially to 3‐hydroxyphenylacetic acid and two to parahydroxyphenylacetic acid, one peak of each attribution overlapped with peaks from other metabolites and so were eliminated from further analysis. Because of the large number of unattributed peaks, ‘unknowns’, in Cluster‐3 (Figure 1), further selection by complementary discrimination for that cluster's phenotypic correlate, pulse rate, was applied (Figure 2A).

Table 2 gives ratios of concentration of the remaining metabolites in those with PD compared to those without. Ratios were essentially unaffected by adjustment for the participant's Bristol Stool Scale consistency rating for that sample: thus, unadjusted concentrations are presented throughout. Overall, metabolite concentrations were significantly lower in PD, with the exceptions of the fermentation‐product ethanol, and two drug‐related metabolites, the dopamine metabolite 3,4‐dihydroxyphenylacetic acid (DOPAC) and unknown‐25. Being prescribed the dopamine precusor levodopa (in oral combination with a peripheral decarboxylase inhibitor, with or without a catechol‐o‐methyl transferase inhibitor) was associated with a large increase in DOPAC [mean (95% CI) 64% (26%, 115%), *p* = .001, adjusted for PD‐status and demographic covariates]. Being prescribed levodopa or a monoamineoxidase‐B inhibitor was associated with an increase in unknown‐25 of 22% (2%, 46%) or 28% (8%, 51%), respectively (*p* = .03 and .004 after adjustment). These drug‐related metabolites were excluded from heatmapping.

**TABLE 2:** Faecal metabolite concentration ratio, with PD to without

Cluster	Metabolite	Ratio (95% CI) concentration PD/remainder, before and after adjustment for stool consistency		
		Before	After	*q* Value†
Cluster‐1	Butyric acid‐1	0.71 (0.55, 0.91)	0.74 (0.57, 0.95)	.03
	Butyric acid‐2	0.74 (0.61, 0.90)	0.76 (0.63, 0.93)	.01
	Butyric acid‐3	0.67 (0.53, 0.84)	0.69 (0.55, 0.87)	.004
	Propionic acid‐2	0.79 (0.66, 0.93)	0.81 (0.68, 0.96)	.02
Cluster‐2	Tryptophan	0.81 (0.73, 0.90)	0.81 (0.72, 0.90)	.002
	Trimethylamine	0.79 (0.68, 0.92)	0.78 (0.67, 0.91)	.01
	Uracil	0.85 (0.75, 0.96)	0.82 (0.72, 0.93)	.03
	3‐Hydroxyphenylacetic acid‐3	0.78 (0.70, 0.87)	0.77 (0.69, 0.87)	.0003
	3,4‐Dihydroxyphenylacetic acid	1.26 (1.08, 1.48)	1.29 (1.10, 1.50)	.01
Cluster‐3	Parahydroxyphenylacetic acid‐2	0.89 (0.82, 0.97)	0.87 (0.80, 0.95)	.03
	Benzoic acid	0.81 (0.73, 0.90)	0.80 (0.72, 0.89)	.0009
	Nicotinic acid‐1	0.72 (0.61, 0.86)	0.75 (0.62, 0.91)	.003
	Nicotinic acid‐2	0.68 (0.54, 0.86)	0.68 (0.53, 0.87)	.006
	Unknown 35	0.80 (0.67, 0.96)	0.81 (0.67, 0.98)	.05
	Homarine‐1	0.77 (0.67, 0.88)	0.77 (0.67, 0.89)	.002
	Homarine‐2	0.71 (0.62, 0.82)	0.73 (0.62, 0.85)	.0003
	Hypoxanthine	0.77 (0.66, 0.89)	0.81 (0.69, 0.96)	.004
	Imidazole‐ring compound	0.70 (0.60, 0.82)	0.74 (0.62, 0.89)	.0004
	Unknown 51	0.69 (0.57, 0.85)	0.71 (0.57, 0.88)	.003
Cluster‐4	Ethanol	1.24 (1.06, 1.45)	1.20 (1.02, 1.42)	.02
	Hydrocinnamic acid	0.89 (0.83, 0.95)	0.87 (0.81, 0.94)	.004
	Glutaric acid	0.84 (0.76, 0.92)	0.83 (0.76, 0.92)	.003
	Unknown 25	1.13 (1.02, 1.26)	1.12 (1.01, 1.25)	.05

†Age, sex, couple‐status and false‐discovery rate adjusted.

### 3.3 Faecal metabolome signatures of disease facets

Figure 2A shows aggregations of metabolite associations with disease facets in three heatmap areas, each area being confined to one cluster.

Area‐1 (within Cluster‐1) showed strong negative associations of short‐chain fatty acids (SCFAs) with transit time through entire colon and its transverse segment. There was an interaction (*p* = .01) between PD‐status and total colonic transit on butyric acid concentration. The lower the butyric acid concentration, the longer was the colonic transit outside PD [–24% (–33%, –14%) per 10 transit‐markers retained, *p* = .001], but not within PD (Figure 2B). A trend (*p* = .06) towards a similar interaction was seen for propionic acid.

**FIGURE 3:**
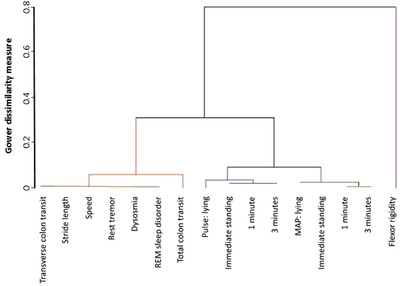
Hierarchical mixed cluster analysis of phenotypic characteristics across diagnostic threshold. Four clusters of phenotypic facets (brown, blue, green and purple) are shown

Area‐2 associates seemingly diverse motor and non‐motor phenotypic facets with deficits in three of the four Cluster‐2 metabolites: tryptophan, trimethylamine and 3‐hydroxyphenylacetic acid (Figure 2A). Area‐2 echoes the negative associations of SCFAs with colonic transit, but slower transit with tryptophan deficit was similar within and outside PD [–8% (–14%, –2%) and –8% (–14%, –3%) per 10 markers retained, respectively, *p* = .01 and .005]. A dendrogram (Figure 3) gives insight into the clustering of phenotypic facets. There were four phenotypic clusters: pseudo‐*T* ^2^ statistic dipped from 56.6 for 4 clusters to 3.8 for 5 clusters. The four were (i) the motor and non‐motor facets of Area‐2; (ii) the pulse series; (iii) the mean arterial pressure series; and (iv) objectively measured flexor rigidity. Rigidity was strikingly dissimilar from the others. Minor dissimilarity was seen between total number of markers retained in colon (which would encompass anal dystonia in PD) and the other Area‐2 facets [markers retained in transverse colon, mean stride length, free walking speed, rest tremor (rated present/absent), dysosmia (present/absent) and rapid‐eye‐movement sleep disorder score].

Area‐3 encompasses the direct negative associations of serial pulse measures with 9 of the 10 Cluster‐3 metabolite peaks (Figure 2A). The strength of association is seen to increases with time standing, but association extends back to the initial lying pulse rate. Cumulative effect of metabolite deficits on pulse could not be estimated because of strong inter‐metabolite relationships. Regarding individual metabolites, benzoic acid concentration was lower by 8.4% (0.4%, 15.7%) per 10 beat increase in pulse, nicotinic acid by 3.7% (0.1%, 7.2%) (*p* = .04 in either case). This was over and above the effect of PD on concentration [–17.7% (–25.9%, –8.5%) and –29.6% (–44.7%, –10.5%), respectively, *p* = .001 and .005]. The single largest metabolite effect was of an imidazole‐ring‐containing compound, with a lower concentration by –10.8% (–15.9%, –5.4%) per 10 beats higher pulse, beside the PD‐effect. Pulse was faster within PD than outside by 5 (2, 8) beats per minute lying, 6 (2, 10) immediate standing, 4 (1, 8) after 1 min, and 4 (1, 8) after 3 min, respectively (*p* = .001, .002, .02 and .02, age and gender adjusted). The faster pulse of PD was irrespective of any postural fall in pressure (Table 1).

### 3.4 Diet in PD and metabolite deficits

**FIGURE 4:**
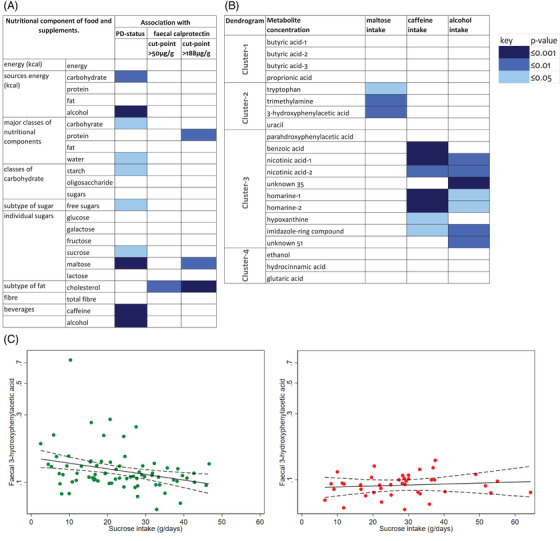
Heatmaps of dietary associations across all participants and a significant effect disease‐status interaction. (A) Nutritional components against PD‐status and faecal calprotectin concentration cut‐points. Median regression. None of following were discriminant for PD: subtypes of fat [saturated, monounstaturated, polyunstaturated (PUFA), cismonounstaturated, cispolyunsaturated, n3PUFA, n6PUFA, trans]; inorganic constituents (sodium, potassium, chloride, calcium, phosphorus, magnesium, iron, zinc, copper, manganese, selenium, iodine); vitamin A (total, retinol, beta‐carotene); vitamin B (thiamine, riboflavin, niacin, pantothenic acid, pyridoxine, biotin, folic acid/folate, cobalamins); other vitamins (C, D, E, K1) and non‐starch polysaccharides. (B) Faecal metabolites according to maltose, caffeine and alcohol intake. (Spearman's rank correlation test). (C) Association of a Cluster‐3 faecal metabolite, 3‐hydroxyphenylacetic acid, with sucrose intake according to PD‐status. Regression lines (95% CI) are shown, data points for participants without PD being in green, with PD in red

Figure 4 shows dietary associations across all participants. Although total energy intake was not higher in PD, that of carbohydrate was (Figure 4A). Intake of starch (*p* = .05) and of the free sugars, sucrose (*p* = .02) and, in particular, maltose (*p* = .001) contributed to this. Total free‐sugar intake was higher by a median (95% CI) of 6.4 (0.4, 12.3) g/day in PD (*p* = .04) (Table 1). Maltose intake was 1.9 (1.2, 3.3) g/day outside PD, higher in PD by 1.2 (0.5, 1.9) g/day (*p* = .001). Caffeine, alcohol and water intakes were lower in PD (*p* = .001, .001 and .05, respectively) (Figure 4A and Table 1). Figure 4B shows the correlations of metabolite concentrations, by Cluster, with a free sugar (maltose taken as exemplar), caffeine and alcohol intakes.

Further analysis showed that maltose intake had a small additional decremental effect on concentrations of Cluster‐2 metabolites [e.g. –5.0% (–9.0%, –0.01%) and –3.1% (–6.2%, –0.00%) per g on trimethylamine and 3‐hydroxyphenylacetic acid, respectively], over and above that of PD [–21.0% (–32.6%, –7.4%) and –22.8% (–31.3%, –13.2%)]. Indeed, these additional effects appeared embedded outside PD [–7% (–13%, –1%) and –5% (–10%, –1%) per g] not within, but the disease‐status interaction did not reach statistical significance. However, Figure 4C shows that for sucrose, the interaction between intake and PD‐status on 3‐hydroxyphenylacetic acid did reach significance (*p* = .02), sucrose having a negative effect on the metabolite concentration outside PD [–8.8% (–14.9%, –2.1%) per 10 g, *p* = .01], with no apparent effect within PD. (Ratio of maltose to sucrose intake was 1/10 overall.)

In contrast, caffeine and alcohol intakes each correlated positively with (7/10) Cluster‐3 metabolites (Figure 4B). That is, although PD had a negative effect on Cluster‐3 metabolites [e.g. –15.6% (–24.4%, –5.9%) and –29.0% (–44.1%, –9.8%) for benzoic and nicotinic acid concentrations, respectively], caffeine appeared to counteract this by a positive relationship [6.7% (1.7%, 11.4%) and 14.2% (2.2%, 27.5%) per 100 mg] irrespective of PD‐status. Similarly, water intake correlated positively, but only weakly so and with a smaller number (3/10) of Cluster‐3 metabolites (parahydroxyphenylacetic acid, homarine and hypoxanthine: *p* ≤ .05 in each case). Water intake also correlated positively with one Cluster‐4 metabolite, glutaric acid (*p* = .005).

### 3.5 Faster pulse: circulating CCL20 complements effect of faecal imidazole deficit

**FIGURE 5:**
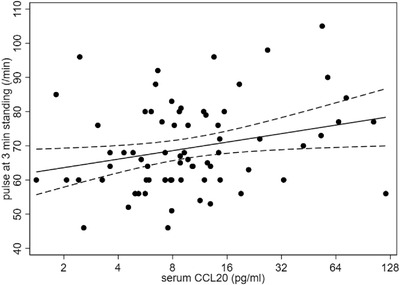
Exploratory analysis: association of pulse rate with serum chemokine (C‐C motif) ligand CCL20 in first 72 participants, irrespective of PD‐status. Regression line (95% CI) is shown. NoteA complementary rise in pulse rate was associated with a deficit in the Cluster‐3 imidazole‐ring metabolite: these effects on pulse together explained 12% of its variance

In this exploratory analysis, none of the 8 serum immune analytes compliant with quality control criteria, discriminated for PD‐status. However, CCL20 had a positive correlation with serial pulse readings (*p* = .03 at 3 min standing) (Figure 5). So, pulse was faster the higher the CCL20 and the greater the deficit in Cluster‐3 metabolites. These effects on pulse were complementary [*p* = .02 and .03 for best individual metabolite predictor (the imidazole‐ring compound) and CCL20 respectively: adjusted *R* ^2^ 12%]. There was a positive correlation between CCL20 and total colonic transit time (*p* < .05), but this did not complement the effect of butyric acid or tryptophan on transit. Faecal calprotectin did not provide insights into the CCL20 associations.

### 3.6 Faecal calprotectin and PD

Age, in isolation, appeared to have a significant cross‐sectional effect on faecal calprotectin concentration (mean (95% CI) increase 24.5% (6.3%, 43.0%) per decade, *p* = .009). Age‐at‐diagnosis of PD had no effect. No within‐participant temporal effects were detected for calprotectin over a mean of 672 (SD 304) days (29 participants with 2 observations). Sex and body mass index had no significant effect. Regarding drug consumption (Table 1), with a PPI, faecal calprotectin was higher by mean (95% CI) 161% (67%, 309%) (*p* < .001, PD‐status adjusted). More participants with PD (21%) than without (9%) were taking PPIs (Fisher's exact test, *p* = .03). Non‐steroidal anti‐inflammatory drug consumption had no effect on calprotectin. Antimicrobial history was associated: overall, calprotectin was higher [by 17% (2%, 35%), *p* = .02] with any antimicrobial courses in the last 3 years, two courses having a large effect [102% (17%, 249%), *p* = .01]. The higher calprotectin was accounted for by exposure within the year preceding the faecal sample. Number of antimicrobial courses was greater with PD than without (*p* = .006), time since the last course similar.

**FIGURE 6:**
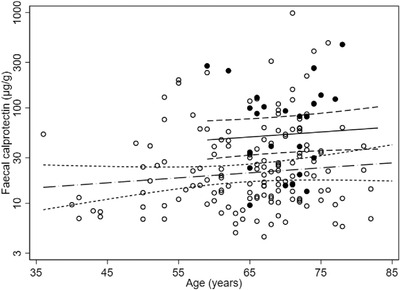
Effect of proton‐pump inhibitor (PPI) exposure on faecal calprotectin concentration. Solid regression line (95% CI) is for participants taking a PPI, dashed regression line for the unexposed, model predictions being as if all participants were without PD. Filled circles represent individuals taking a PPI and open circles the unexposed

Taking all available measurements, calprotectin was significantly higher in PD by 61% (18%, 120%) (*p* = .003, Table 1). The effect remained [44% (5%, 98%)] after adjustment for age, PPIs and antimicrobial exposure, only the additional effect of PPIs reaching significance (*p* = .001). Figure 6 shows that any ageing effect on calprotectin concentration is confounded by iatrogenic inflammaging, namely PPI exposure. The odds of having a concentration compatible with inflammation (>50 μg/g)^40^ were doubled in PD: 2.07 (0.97, 4.45) (*p* = .06). Indeed, 16% of PD‐probands exceeded the (188 μg/g)^41^ cut‐point adopted for ‘clinically significant inflammation’ comparable with that found in IBD, on at least one occasion, whereas only 2% (2/87) of the remainder did (Fisher's exact test, *p* = .004). However, the likelihood of a second calprotectin result concurring with first in PD was slight with respect to the lower cut‐point [Kappa (SE) 0.04 (0.18)], nil with respect to higher. Calprotectin tended to be higher with greater severity of PD (UPDRS total motor score^28^) and worse functionality (Hoehn and Yahr staging) (*p* = .1 and .09, respectively). Being a proband's partner, rather than a control‐proper, had no significant effect on either the odds of exceeding the lower cut‐point [mean (95% CI) 1.36 (0.47, 3.93)], or on the calprotectin concentration itself [5% (–28%, 51%)] using all measurements and adjusting for PPI prescription.

### 3.7 Diet in PD and intestinal inflammation

Of the dietary components discriminant for PD (Figure 4A), only maltose was associated with faecal calprotectin. Where the higher ‘clinically significant inflammation’ cut‐point was exceeded, maltose intake was markedly higher, by a mean (95% CI) of 2.27 (0.42, 4.11) g/day (*p* = .02), irrespective of PD‐status. Overall, calprotectin tended (*p* = .07) to be higher by 12% (0%, 26%) per gram of daily maltose intake, after PPI adjustment. There was some evidence for a predisposition or sensitisation to maltose‐related intestinal inflammation in PD (maltose intake by PD‐status interaction on calprotectin concentration, *p* = .1): a 23% (4%, 45%) increase in calprotectin per gram of daily maltose within PD (*p* = .02, PPI adjusted), no increase outside.

Cholesterol and protein intakes related to calprotectin, but not to PD (Figure 4A). Over all participants, calprotectin tended to be higher by 19% (0%, 43%) per 100 mg of daily cholesterol intake (*p* = .07), the median (interquartile range) intake being 220 (165, 280) mg/day. Where the lower or higher calprotectin cut‐points were exceeded, cholesterol intake was higher by 64 (14, 115) or 135 (54, 216) mg/day, respectively (*p* = .01 and .001). A significantly higher protein intake was associated with exceeding the higher cut‐point, but it did not contribute to a model explaining increase in calprotectin in terms of maltose plus cholesterol intakes [17% (4%, 31%)/g and 20% (0%, 44%)/100 mg, respectively (*p* = .008 and *p* <.05)]. After PPI adjustment, the size of these effects was 15% (3%, 28%)/g and 16% (–3%, 38%)/100 mg.

## 4 DISCUSSION

### 4.1 Summary

We are the first to use an untargeted approach to explore the complete NMR faecal metabolic profile, according to PD‐status, and then cross‐examine discriminant metabolites against measures of disease facets across the diagnostic divide. The approach was rewarding in that the deficits in 3 of the 4 faecal metabolite clusters had clear disease‐facet profiles, and the nature of the metabolites gave clues to potential underlying mechanisms. Metabolite clustering corresponded to well‐recognised dietary characteristics of PD, lower caffeine, alcohol and water intakes and a new finding of increased free‐sugar intake, especially of maltose. Effects of diet on metabolome might be via reshaping the microbiota, rather than direct. The excess intake of free sugars was also linked to the intestinal inflammation of PD, as measured by calprotectin.

Drivers of the pathophysiology may operate before and/or beyond the diagnostic threshold of this pleomorphic disease. Defining windows of opportunity for disease modification is the goal. Although a marked deficit in faecal SCFAs was seen in PD, the expected (from rodent models)^45^ inverse correlation of butyric acid concentration with prolonged colonic transit was evident only outside PD. However, irrespective of diagnostic threshold, a phenotypic cluster containing delay in colonic transit (plus brady‐/hypokinesia, tremor, dysosmia and rapid eyeball movement sleep disorder, but not rigidity) correlated inversely with concentrations of the tryptophan‐containing metabolite cluster.

### 4.2 Slow colonic transit and deficit in faecal SCFA and tryptophan metabolite Clusters

Slow colonic transit may encourage a bacterial microbiota deficient in taxa whose metabolites are essential to gut health and motility, a vicious cycle being set‐up. Instigation (e.g. by a prokaryotic‐virus or phage^42^, ^46^ that persists) may be decades before the presentation. Serotonin increases gastrointestinal motility: its precursor tryptophan is in deficit in the faecal metabolome of PD. SCFAs are also in deficit in the PD faecal metabolome: they promote the tryptophan‐hydroxylase required for serotonin synthesis in enterochromaffin cells. Tryptophan deficiency is characterised by T‐cell and tight junction dysregulation.^47^ Colonic production of SCFAs is a major source of nutrition for the epithelium.^48^ In our PD cohort, there was no shortage of fibre substrate for microbial fermentation to SCFAs, compared with outside PD. Insufficient microbial enzymes for fermentation could underlie the relative deficit in PD. Reduced faecal abundance of the *Lachnospiraceae* family, notable SCFAs producers, is reported in PD,^49^, ^50^ but other SCFA‐producers are reported as increased.^50^

### 4.3 Potential *forme fruste* of SIRS

Far from the concept of cold neurodegeneration, the parkinsonian syndrome may be a *forme fruste* of SIRS. Pulse rate was faster in PD, irrespective of postural fall in blood pressure, and despite any cardiac sympathetic neuropathy. Future work will include measurement of tympanic temperature. Pulse was faster with faecal deficits in anti‐fungal molecules (benzoic acid and an imidazole‐ring compound);^51^, ^52^ an anti‐inflammatory (nicotinic acid, a form of vitamin B3);^53^, ^54^ a barrier function protector (hypoxanthine);^55^ and an osmolyte, maintaining cell integrity and influencing protein‐folding (homarine).^56^

Dietary intake of vitamin B3 was not lower in PD, but its deficiency has been reported with consumption of levodopa combined with decarboxylase inhibitors.^57^ Decarboxylase inhibitors reduce B3 synthesis from tryptophan by intestinal bacteria.^57^ B3 has high affinity for an anti‐inflammatory receptor GPR109A, which is upregulated in PD.^58^ In a mouse model, B3–GPR109A signalling induces differentiation of regulatory T (Treg) cells and suppresses colitis.^59^ GPR109A agonists suppress lipopolysaccharide‐induced gut inflammation through NFκB inhibition.^58^ Indeed, the SCFA GPR109A‐agonist, butyrate, reduces inflammation in CD by this mechanism. Moreover, in PD, there are biological gradients of worsening motor scores on blood mononuclear cell NFκB expression.^19^

Pulse was also faster with a higher serum CCL20 (chemotactic for lymphocytes/dendritic cells towards mucosal epithelium).^60^ Indeed, there were complementary associations of faecal metabolite and serum chemokine concentrations with pulse, irrespective of diagnostic threshold. CCL20 is the only known ligand for the CCR6 chemokine receptor. Pro‐inflammatory Th17 and Treg cells, in the intestine and mesenteric lymph nodes, express CCR6, as do dendritic cells. Disruption in the CCL20‐CCR6 axis, with disordered regulation of Th17 and Treg cells, is proposed in IBD. Here, as regards a mediator role, we link serum CCL20 with prolonged colonic transit, as well as a higher pulse. CCL20 expression is reported to depend on IL‐6 amplifier activation:^61^ future deeper modelling of its role may benefit from incorporation of IL‐6 as a covariate. Replacing critical metabolites, directly or by microbiota modification, may be beneficial. Surrogacy in their dietary correlates should be considered. Reduced caffeine intake could be surrogate for water shortfall, or vice versa, intolerance of alcohol in PD a consequence of increased colonic production.

### 4.4 Premature and iatrogenic inflammaging

The increase in serum IL6 in PD, over and above the ageing effect,^17^ is a classic example of premature ageing. Faecal calprotectin provides evidence of iatrogenic inflammaging in PD.^16^, ^17^ Calprotectin appeared to increased by 25% per decade of age over all participants, the higher concentration in PD approaching the equivalence of two decades of ageing. However, the ageing effect was confounded by PPI exposure.

Follow‐up suggests a relapsing/remitting pattern of intestinal inflammation, even where initial calprotectin concentration was at a level comparable with that of IBD. Extensive longer‐term follow‐up, mindful of PPI consumption, may reveal heterogeneous patterns fitting with those of PD (e.g. ‘unremitting progression’ and ‘burn‐out’).

### 4.5 Intestinal inflammation as a ‘half‐way‐house’ to neuroinflammation

Reducing the gross excess in maltose intake in PD might ameliorate clinically significant intestinal inflammation. Any predisposition to maltose‐related intestinal inflammation in PD might be explained by reduced abundance of bacterial taxa which catabolise free sugars.^49^ Reducing free‐sugar intake provides a potential opportunity for prophylaxis by boosting the tryptophan‐containing metabolite cluster. Dietary cholesterol, and antimicrobial exposure (≥2 courses in last 3 years) and PPI‐usage are potential provocateurs of intestinal inflammation, in ascending order of potency. PPIs cause increased susceptibility to enteric infections, and an increase in both bacteria typical of the mouth and potential pathogens, in the context of overall decrease in faecal bacterial richness.^62^ More alterations in gut microbiota were associated with PPIs than with antimicrobials. The potential importance of medicinal provocateurs resonates in PD, where there is a cumulative increase in rigidity with successive antimicrobial courses.^25^ Monitoring the effect of chronic PPI‐usage on incidence and progression of PD might corroborate intestinal inflammation as a ‘half‐way‐house’ to neuroinflammation.

An alternative source of inflammation to colitis is small intestine bacterial overgrowth (SIBO). However, faecal calprotectin is not a reliable marker of small intestinal inflammation: patients with ileal CD may not have markedly elevated concentrations.^63^ Previously, we found SIBO in two‐thirds of PD‐probands on lactulose hydrogen breath testing. This was accompanied by higher circulating natural killer (NK) and CD4+ lymphocyte counts, and lower (as if sequestrated in gut) neutrophil counts.^20^ Flexor rigidity and hypokinesia were associated with higher circulating NK and CD4+ counts (CD4+ apparently modulating NK‐effect on rigidity), tremor with a lower neutrophil count. Overgrowth in PD is likely to result from reflux of microbiota from a stagnant ascending colon.^1^ Indeed, the higher prevalence of hydrogen breath test positivity with a lactulose substrate than when using the absorbable sugar, glucose, may represent distal colonisation.^13^

### 4.6 Comparisons with other published work

Our systematic review^10^ firmly established the inter‐relationship of PD with intestinal inflammation and bacterial translocation, evidence for increased permeability (non‐mediated passive diffusion) and loss of integrity being limited. A direct role of a pro‐inflammatory gut microbiota, specific pathogens, or pathogen‐associated molecular patterns (PAMPs)^15^ in driving PD is not investigated here. However, our observational finding that intervention with maintenance bulk, osmotic and/or enterokinetic laxatives appeared to stem the, on average, 6% year‐on‐year, increase in rigidity, is compatible with a role of dysbiosis in the pathogenesis.^26^ Previous untargeted NMR metabolomic approaches have concentrated on blood, saliva and urine,^64^ one on faeces.^65^ The deficit in faecal SCFAs in PD is in agreement with the latter and three SCFA‐targeted studies.^66^, ^67^, ^68^

### 4.7 Limitations and crucial future research directions

Regarding limitations, this is just a step on the way to a multidimensional fully quantifiable pathophysiological model for PD and overlap diseases, to describe where an individual is now and the trajectories with time and interventions. Candidate sections of the ‘drivers and mediators of PD’ jigsaw are assembled in a gastroenterological framework. The purpose is to lay a foundation for classification modelling for disease‐status, description of transitions in disease spectrum and development of time‐series models to understand evolution‐related factors. The next step is incorporation of faecal microbial taxonomy and functionality and the human genetics of immunoinflammatory response and PD‐risk. An adaptive approach allows expansion, in terms of cohorts of particular interest and datasets [e.g. reprioritise our reference list of serum immune analytes (see Supporting Information), initially selected on a 3‐study criterion as discriminant for PD^39^]. The comparator group included probands’ nominees: this may result in a group more alike, in some characteristics, to probands than had they been randomly selected. Ignoring this design feature in the analysis could have introduced a conservative bias in magnitude of effects (i.e. towards the null hypothesis).

## 5 CONCLUSIONS

In conclusion, it is essential for comprehensive identification of disease modification targets to consider the phenotypic facets as having different drivers and mediators, and to measure facets irrespective of diagnostic threshold. We assemble two potential pathophysiological pathways (a vicious cycle of colonic delay and deficit in beneficial bacterial metabolites; a halfway house of intestinal inflammation) and give indicative evidence for a third (a pro‐inflammatory microbiota). Scope for simultaneous or sequential interplay between pathways is recognised. Disease‐modifying interventions, with reference to optimal timing in the natural history (e.g. premature/accelerated inflammaging or SIRS), site (small intestine or colon) and target (microbe, PAMPs, metabolite or inflammation), are needed. Simple good‐practice measures are potentially disease‐modifying: (i) reducing candidate provocateurs of intestinal inflammation, dietary (maltose and cholesterol) and, where appropriate, medicinal (PPIs, antimicrobials), and (ii) rigorously treating slow colonic transit with hydration and maintenance non‐irritant laxatives.

## CONFLICT OF INTEREST

The authors are not aware of financial conflicts with the subject matter or material discussed in this article with any of the authors or their academic institutions or employers.

## Supporting information

Metabolite annotation; Serum immune analytes

## Data Availability

All data associated with this study are presented in the manuscript or the supplementary information. Additional files are available in figshare at (for 1D spectra) https://figshare.com/articles/dataset/Raw_dataset_Sylvia_Dobbs_leaky_gut_zip/20146637 and (for 2D spectra) https://figshare.com/articles/dataset/201027_Aisha_fecal_water_2Ds/20146877.
