# Supplementary material for: Faecal metabolite deficit, gut inflammation and diet in Parkinson's disease: Integrative analysis indicates inflammatory response syndrome
Source: Clin Transl Med. 2023 Jan 1;13(1):e1152. doi: 10.1002/ctm2.1152 (PMC9806009; doi:10.1002/ctm2.1152)
Supplement: Supplementary file 1 — Metabolite annotation; Serum immune analytes [file CTM2-13-e1152-s001.docx]

**SUPPORTING INFORMATION**

**Supplementary Methods**

***Metabolite annotation***

The strategy of annotation described is comprehensive with a view to providing a large number of annotated metabolites at the Metabolomics Standards Initiative level of annotation of 2 (matching to the database spectral data).^1^ The COLMAR website^2^ was used to generate a first list of potential metabolites, using both the heteronuclear single quantum coherence (HSQC) and the total correlation spectroscopy (TOCSY) spectra. Then the identification of each metabolite in this first list was done manually using the TOCSY and HSQC spectra, by comparison with the spectra in libraries (see **2.4 Faecal metabolome**).

Metabolite annotation was thus achieved using a combination of strategies (Table S1). On-line and in house reference databases were used to assign the chemical shifts to metabolites. Standard 1D ^1^H NMR pulse sequence with water peak presaturation and 2D NMR methods [such as 2D J-resolved, 2D ^1^H-^13^C heteronuclear single quantum coherence (HSQC); ^1^H-^1^H total correlation spectroscopy (TOCSY)] were applied to the selected or pooled samples to confirm the signals and their chemical shifts. Some metabolites were confirmed by spike-in experiments using authentic chemical standards.

Some metabolites, which contributed to the statistical modelling (e.g. unknown 49 in Cluster-3) remained unannotated. Its broad singlet peak detected at 8.414-8.435 ppm in 1D ^1^H NMR spectra was annotated as imidazole-containing compound based on its shape and chemical shift values, and similarity to ^1^H and ^13^C NMR signals of histidine and related metabolites (Figure S1A and S1B, respectively). The spike-in experiments with L-histidine, 1-methylhistidine, 3-methylhistidine, imidazolacetic acid, and imidazolepropionic acid proved that although the unknown signal could not be assigned to one of the tested metabolites, it has similar spectral characteristics (Figure S1A and S1B). Namely, the signal of the unknown compound and spiked in metabolites is a broad peak sensitive to pH value. Further metabolite identification work is required to reveal the structure of this metabolite.

***Serum Immune analytes***

Multiplex-assays were used for 22 of 23 serum immune-analytes, associated in literature with PD and its co-morbidities: chemokine (C-C motif) ligand (CCL3); CCL4; CCL20; C-X-C motif chemokine ligand (CXCL)10; epidermal growth factor (EGF); granulocyte colony-stimulating factor (G-CSF); granulocyte-macrophage colony-stimulating factor (GM-CSF); growth hormone; intercellular adhesion molecule (ICAM)-1; interferon (IFN)-γ; interleukin (IL)-1β; IL-2; IL-4; IL-5; IL-6; IL-10; IL-13; leptin; macrophage migration Inhibitory factor (MIF); resistin; transforming growth factor (TGF)-α; tumour necrosis factor (TNF)-α. A sandwich enzyme-linked immunosorbent assay was used for assay of the 23^rd^ serum immune-analyte, IL-17. Samples from the first 72 participants were assayed in duplicate, in an exploratory mission.

**Table S1**. **Chemical shifts annotated and annotation strategy for each ^1^H NMR faecal metabolite**.

| **Cluster** | **Metabolite** | **Chemical shift, ppm (multiplicity*)** | **Confirmed by** |
| --- | --- | --- | --- |
| Cluster-1 | butyric acid-1 | 0.88-0.91 (t) | 1D & 2D NMR (TOCSY, HSQC), comparison to spectral databases |
|  | butyric acid-2 | 1.52-1.59 (m) | 1D & 2D NMR (TOCSY, HSQC), comparison to spectral databases |
|  | butyric acid-3 | 2.145-2.17 (t) | 1D & 2D NMR (TOCSY, HSQC), comparison to spectral databases |
|  | propionic acid-2 | 2.18-2.21 (q) | 1D & 2D NMR (TOCSY, HSQC), comparison to spectral databases |
| Cluster-2 | tryptophan | 7.52-7.55 (d) | 1D & 2D NMR (TOCSY), comparison to spectral databases |
|  | trimethylamine | 2.89-2.9 (s) | 1D & 2D NMR (HSQC), comparison to spectral databases |
|  | uracil | 5.79-5.82 (d) | 1D & 2D NMR (TOCSY, HSQC), comparison to spectral databases |
|  | 3-hydroxyphenylacetic acid-3 | 6.67-6.71 (d) | 1D & 2D NMR (TOCSY), comparison to spectral databases |
|  | 3,4-dihydroxyphenylacetic acid | 7.22-7.26 (d) | 1D & 2D NMR (TOCSY), comparison to spectral databases |
| Cluster-3 | 4- hydroxyphenylacetic acid-2 | 7.16-7.19 (d) | 1D & 2D NMR (TOCSY, HSQC), comparison to spectral databases |
|  | benzoic acid | 7.85-7.89 (d) | 1D & 2D NMR (TOCSY), comparison to spectral databases |
|  | nicotinic acid-1 | 8.60-8.63 (dd) | 1D & 2D NMR (TOCSY, HSQC), comparison to spectral databases |
|  | nicotinic acid-2 | 8.93-8.95 (d) | 1D & 2D NMR (TOCSY, HSQC), comparison to spectral databases |
|  | unknown 35 | 6.363-6.388 (d) |  |
|  | homarine-1 | 7.943-7.988 (m) | STOCSY, 1D & 2D NMR (TOCSY, HSQC), comparison to published data, ^1^H NMR spike |
|  | homarine-2 | 8.025-8.055 (d) | STOCSY, 1D & 2D NMR (TOCSY, HSQC), comparison to published data, ^1^H NMR spike |
|  | hypoxanthine | 8.179-8.197 (s)  8.20-8.22 (s) | 1D & 2D NMR (HSQC), comparison to spectral databases, ^1^H NMR spike |
|  | Imidazole-containing compound (unknown 49) | 8.414-8.435 (s) | STOCSY, 1D & 2D NMR (HSQC), ^1^H NMR spike |
|  | unknown 51 | 8.52-8.56 (s) |  |
| Cluster-4 | ethanol | 1.14-1.21 (t) | 1D & 2D NMR (TOCSY, HSQC), comparison to spectral databases |
|  | hydrocinnamic acid | 2.47-2.51 (t) | 1D & 2D NMR (TOCSY, HSQC), comparison to spectral databases |
|  | glutaric acid | 1.77-1.81 (m) | 1D & 2D NMR (TOCSY), comparison to spectral databases |
|  | unknown 25 | 3.12-3.15 (s) |  |

*s, singlet; d, doublet; dd: doublet of doublet; t, triplet; q, quartet; m, multiplet


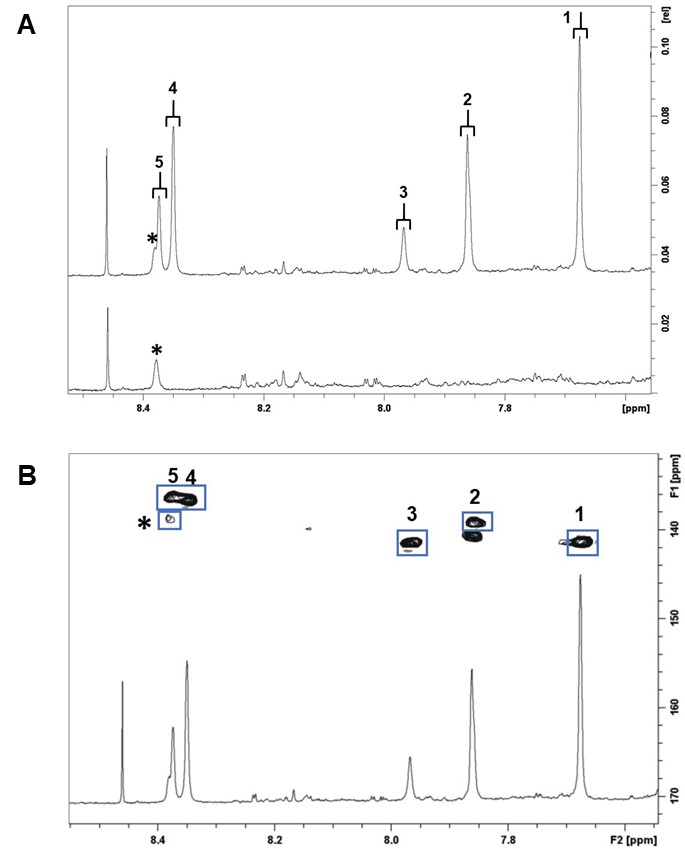


**Figure S1.** (**A**) 600 MHz 1H NMR spectra of a selected faecal sample before (lower spectrum) and after (upper spectrum) spiking authentic chemical standards of imidazole-containing metabolites. (**B**) ^1^H-^13^C 2D HSQC spectrum overlaid with 1D 1H NMR spectrum with highlighted cross peaks of the unknown compound and spiked in authentic chemical standards. **(*)** unknown 49 imidazole-containing compound; **(1)** 1-methylhistidine; **(2)** histidine; **(3)** 3-methylhistidine; **(4)** imidazolacetic acid; **(5)** imidazolepropionic acid.

**References**

1. Sumner LW, Amberg A, Barrett D, et al. Proposed minimum reporting standards for chemical analysis Chemical Analysis Working Group (CAWG) Metabolomics Standards Initiative (MSI). *Metabolomics* 2007;3:211-221.

# Bingol K, Li D-W, Zhang B, *et al*. Comprehensive Metabolite Identification Strategy Using Multiple Two-Dimensional NMR Spectra of a Complex Mixture Implemented in the COLMARm Web Server. Analytical Chemistry **2016;**88:12411-12418.
